# Autoimmune Hepatitis as a Unique Form of an Autoimmune Liver Disease: Immunological Aspects and Clinical Overview

**DOI:** 10.1155/2012/312817

**Published:** 2012-12-12

**Authors:** Hind I. Fallatah, Hisham O. Akbar

**Affiliations:** ^1^Medical Department, Arab Board and Saudi Board of Internal Medicine, MACP, King Abdul Aziz University Hospital, P.O. Box 9714, Jeddah 21423, Saudi Arabia; ^2^King Abdul Aziz University Hospital, Jeddah, Saudi Arabia

## Abstract

Autoimmune hepatitis (AIH) is a unique form of immune-mediated disease that attacks the liver through a variety of immune mechanisms. The outcomes of AIH are either acute liver disease, which can be fatal, or, more commonly, chronic progressive liver disease, which can lead to decompensated liver cirrhosis if left untreated. AIH has characteristic immunological, and pathological, features that are important for the establishment of the diagnosis. More importantly, most patients with AIH have a favorable response to treatment with prednisolone and azathioprine, although some patients with refractory AIH or more aggressive disease require more potent immune-suppressant agents, such as cyclosporine or Mycophenolate Mofetil. In this paper, we discuss the immunological, pathological and clinical features of AIH, as well as the standard and alternative treatments for AIH.

## 1. Introduction

Autoimmune hepatitis (AIH) is a chronic, immunologically mediated inflammatory liver disorder of unknown etiology [1–3]. It is characterized by the presence of high levels of circulating autoantibodies, hypergammaglobulinemia, and elevated levels of serum transaminases [1–3]. AIH was first recognized in 1942 by Amberg [4]. In 1950, Waldenstr Öm described a form of chronic hepatitis in young females that was associated with jaundice, elevated levels of serum immunoglobulins, and amenorrhea [5]. Several different names have been ascribed to this condition, including plasma cell hepatitis and lupoid hepatitis [6, 7], but Mackay et al. designated this condition as autoimmune hepatitis in 1965 [8]. Compared to other forms of autoimmune liver disease (AILD), AIH is characterized by a hepatocellular pattern of elevated levels of liver enzymes, positive tests for non-disease-specific antinuclear antibodies (ANA) and/or smooth muscle antibodies (SMA), which are the histological hallmark of AIH interface hepatitis on liver biopsy, and an optimal response to steroids in most patients [9–11].

## 2. Epidemiology

AIH is the cause of 11–20% of all cases of chronic hepatitis in Western countries [12, 13] and demonstrates a disease prevalence of 1 : 5,000–1 : 10,000 and an incidence of 0.85/100,000 in developed countries [9, 11, 14, 15]. In countries that are endemic to viral hepatitis, AIH is less commonly recognized than chronic viral hepatitis [9, 16–19]. Females are more often affected because 70–80% of AIH patients are generally female [11, 20–24]. In our report on AIH, 75.7% of the patients were females [18]. Although AIH typically affects younger individuals, approximately 20% of AIH patients are diagnosed after the age of 60 [11, 15, 18, 20]. AIH type 1, which is more common than AIH type 2, mainly affects adolescent and adult females (the female-to-male ratio is 4 : 1) [9, 10, 18, 20, 21, 24–26]. However, AIH type 2 predominantly affects children younger than 18 years of age with a female-to-male ratio of 9 : 1 [9, 25, 27–29]. AIH has a variable onset age, which differs according to the geographic distribution and the ethnic group. For instance, Japanese AIH patients demonstrate an onset age of 50 years, whereas Caucasian patients typically demonstrate an onset age of 10–20 years [17, 30, 31]. Similarly, our report on AIH in Saudi Arabia and other reports from India have shown a younger onset age in these populations compared to other Asian countries, the US, and Europe [9, 18, 20, 21, 32–34].

## 3. Etiology and Risk Factors

AIH is characterized by the loss of immune tolerance to antigens that are present on hepatocytes, as well as by impaired immune regulation [11, 35]. No clear etiological factor has been identified for the initiation of the immune-mediated damage to the liver tissue that is observed with AIH, but many triggering risk factors have been suggested to play a role in the initiation of this immunologically mediated liver injury. Genetic predisposition is also thought to play a major role in the development of AIH.

## 4. Genetic Risk Factors

The major histocompatibility complex (MHC), which is also known as human leukocyte antigen (HLA), is located on chromosome 6 and is the most commonly reported gene associated with AIH [11, 26, 32, 35, 37, 38]. The DRB polypeptide of the MHC class II that presents antigen to CD4+ T lymphocytes has been the most extensively studied MHC component. In the US and Europe, AIH susceptibility is associated with the HLA-DRB1*0301(DR3) and HLA-DRB1*0401(DR4) alleles [11, 17, 32, 37, 39]. Moreover, older patients have been shown to have a higher frequency of HLA-DRB1*04 than young adults or children, [11, 17, 37]. In addition, North American and European children with AIH type 2 frequently carry the HLA-DRB1*03 allele [11, 17, 38]. Based on reports on Japanese cases of AIH, which constitute the most extensively studied population of AIH patients in Asia, carriers of HLA-DRB1*0301 have been shown to be extremely rare [40]. In fact, AIH among Japanese and Argentine adults is more commonly associated with the DRB1*0405 allele (DR4). This difference in the HLA gene association between Japanese and Western populations might explain the difference in the age of onset of AIH between the two populations [38, 41, 42]. Furthermore, in Caucasians, HLA-DR*β*1*1501 is associated with protection from AIH [42], whereas this gene is associated with a weak susceptibility instead of resistance to AIH in Japanese patients [26]. Among Mexican individuals with AIH, the predominant allele is DRB1*0404 (DR4) [34], whereas the predominant allele in Brazilian patients is HLA-DRB1*07 [43]. 

## 5. Triggering Factors

Antigens that are most likely triggered by autoantigen mimicry are thought to play a role in the initiation of the immune system-mediated damage that is associated with AIH [11, 26, 35]. Infections with hepatitis viruses A, B, and C, Epstein Barr virus, and herpes simplex virus have also been implicated as potential viral triggers [26, 44, 45]. Hepatitis C virus is commonly associated with immunological features that are similar to those of AIH; this results in diagnostic difficulties with some hepatitis C virus-infected patients [46, 47]. Several medications, such as atorvastatin [48], diclofenac, isoniazid methyldopa, minocycline, nitrofurantoin, and propylthiouracil, as well as the hepatitis A vaccine [26, 49–51], have also been reported to trigger AIH. Björnsson et al. found that AIH represents 9.2% of all of the cases of drug-induced liver injury (DILI) that were identified in the Mayo clinic over 10 years [50]. AIH can be a feature of recurrent DILI due to exposure to a different drug, as reported by Lucena et al. [51] More recently, the onset of drug-induced AIH caused by the antitumor necrosis factor *α* agents adalimumab and infliximab has been demonstrated [52–54]. It is important to differentiate between drug-induced AIH and immune-mediated drug-induced liver injury because short-term treatment is usually required for the former but withdrawal from the causative drug is sufficient to treat the latter [50]. However, some patients with DILI have exhibited recurrent AIH despite cessation of the causative drug, which suggests that DILI can unmask true AIH [55]. Furthermore, cirrhosis is present in 20% of AIH patients at baseline, but none of the patients with AIH caused by DILI showed cirrhosis at baseline [50]. Moreover, drug-induced AIH may be underrecognized because the causative drug is frequently withdrawn without any additional workup. Furthermore, the coexistence of an infectious agent and a drug, which simultaneously act as triggering factors, was suggested in a case report by Kamiyama et al. [56]. Several environmental agents, such as herbal triggers, have also been reported to initiate AIH, including black cohosh, syo-saiko-to; and Chinese herbal tea [57–61]. Khat, which is commonly chewed by the populations of Yemen and Somalia as a common social habit, is another hepatotoxic environmental agent that has recently been recognized as a triggering agent for a severe form of AIH in young males in these countries [62–65]. In our report of 3 male patients with khat-induced AIH, all of the patients exhibited the classical biochemical and immunological features of AIH; they also all had a favorable response to steroids [62]. We recently had another patient with similar features and decompensated cirrhosis who exhibited a complete biochemical response (normal ALT) to treatment with prednisolone and azathioprine. Thus, even with the variety of risk factors that has been proposed, the specific underlying cause for AIH remains unknown [11]. 

## 6. Immunological Features of AIH

### 6.1. Immune Pathogenesis and the Immunological Aspects of AIH

The immune response that targets the liver in AIH involves cytotoxic T lymphocytes, which damage the hepatocytes via the production of interleukins (IL-2, IL-12, and tumor necrosis factor-a (TNF-a)). However, the molecular target of the T lymphocyte response has not yet been identified [66, 67]. Several studies have demonstrated that the activity of cytotoxic T lymphocytes is greater in AIH patients with active disease, whereas activated T cells are only observed in 40% of the patients in remission [26]. Furthermore, polymorphism of the gene encoding the cytotoxic T-lymphocyte antigen-4 (CTLA-4) on chromosome 2q33 is more common in patients with type 1 autoimmune hepatitis and may represent a susceptibility allele for AIH [68]. CTLA4 is expressed by regulatory T cells (Tregs) and is an essential factor for the negative regulation of the immune response [69]. Another mechanism of immune pathogenesis is the antibody-mediated cellular cytotoxicity, which is initiated by CD8 T cells (the predominant cells found in areas of hepatic inflammation). They recognize the MHC-autoantigen complex on Kupffer cells or hepatocytes [67]. A reduction in the number and proliferative activity of Tregs in response to stimulation has been reported in patients with AIH [70, 71]. Moreover, the activity of natural killer cells, which are known to play a major role in the regulation of the immune response, might be reduced in AIH [11, 72]. More recently, Th17 cells have been shown to play a role in human autoimmune diseases, including AIH [11]. The induction of AIH in animal models has been difficult [73], although Tu et al. recently demonstrated an animal model of AIH in which the antibody-initiated complement activation plays a role in the induction of hepatocyte injury both *in vitro* and *in vivo* [74].

### 6.2. Autoantibodies in AIH

The presence of autoantibodies is one of the distinguishing features of AIH. Immunofluorescence (IFL) is the main technique that is currently used to screen for the autoantibodies that may be diagnostically relevant to liver disease [75, 76].

#### 6.2.1. Anti-Nuclear Antibody (ANA)

ANAs exhibit nuclear staining in the kidney, stomach, and liver because ANAs are specific for nuclear antigens (first described by Miescher and Fauconnet in 1954) [77]. Although no specific ANA nuclear antigen has been identified in AIH type 1 [75], a variety of nuclear molecular targets have been detected, including centromeres, histones, double-stranded DNA, chromatin, and ribonucleoprotein complexes [78]. Moreover, no single ANA pattern is dominant in AIH, although a homogenous pattern is typical [79]. In addition, ANAs are not specific to AIH because ANA staining can also be observed in patients with chronic viral hepatitis B and C [80]. ANAs are also detected in patients with drug-induced hepatitis [13, 50, 51, 55] and other nonliver disorders. However, ANAs represent the sole marker of AIH disease in 13–15% of the patients [78, 81].

#### 6.2.2. Smooth Muscle Antibody (SMA)

SMAs were first described in patients with autoimmune liver disease in 1965 [82]. Since then, these have also been detected in the kidney, stomach, and liver [29, 83]. These antibodies target arterial vessels (V), the glomerular mesangium (G), and the fibers surrounding the kidney tubules (T), which results in the characteristic VGT pattern that is observed with AIH type 1 [84]. The VGT pattern of SMA also targets F-actin (microfilaments or filamentous actin) and the intermediate filaments (vimentin and desmin) [10, 75]. However, 20% of the SMA-positive cases of AIH type1 do not exhibit the typical F-actin/VGT pattern [85], which implies that the absence of this pattern cannot exclude the potential diagnosis of AIH type 1 [85]. However, the IFL SMA pattern is either absent or extremely rare in conditions other than AIH type 1 [86, 87]; thus, this pattern provides a high level of specificity and selectivity [79]. However, the SMA/F-actin pattern can be detected in patients with viral hepatitis and other autoimmune liver disorders [86, 87]. Approximately 35% of all AIH type 1 cases demonstrate SMA positivity alone [78, 81], whereas 55% to 60% of the cases demonstrate both ANA and SMA positivity [78, 81]. Furthermore, although the ANA and SMA levels decrease after immune-suppressive therapy [78], neither level has been shown to correlate with the response to therapy. In addition, neither level has have been found to be associated with the clinical or histological disease severity of AIH type 1 [88].

#### 6.2.3. Anti-Actin Antibodies

Studies have shown that a subset of patients who are positive for SMAs also demonstrate F-actin staining [89, 90]. These markers are of greater prognostic value and have been associated with a poor response to treatment with corticosteroids [91–93]. Czaja et al. showed that liver-related death was reported in 6% of the anti-actin-positive patients compared with 0% of the anti-actin-negative patients [91]. Similarly, the same report described that 10 out of the 11 studied patients who had liver transplantation were anti-actin positive and seropositive for SMA antibodies [91]. 

#### 6.2.4. Anti-Soluble Liver Antigen (SLA)

The anti-soluble liver antigen auto-antibodies were first described by Manns et al. in 1987 [94]. These antibodies were also shown by Berg et al. to serve as markers of the pancreas and have been described as anti-liver pancreas antibodies (LP) [95]. SLAs are directed against tRNAs and can be detected in 47.5% of the AIH patients. These antibodies are highly disease specific for severe forms of AIH type1 and are typically associated with a fatal outcome [96]. SLAs are also associated with the presence of HLA DRB1*0301 [97]; this HLA association has been shown to predict the development of severe forms of AIH, as well as the relapse rate following treatment cessation and a greater likelihood of liver disease-related mortality [10, 97–99]. SLAs are mainly identified in patients who are also positive for ANAs and either SMAs or LKM-1. However, SLAs are also detected in isolation in up to 20% of AIH cases [100, 101]; therefore, in these cases, SLA detection is an important component of the AIH diagnosis [98]. 

#### 6.2.5. Liver-Kidney Microsomal Antibody (LKM-1)

The LKM-1 antibodies were first described in 1974 by Rizzetto et al. [102] The target antigen of LKM-1 antibodies is cytochrome P450 2D6 (CYP2D6) [1, 3, 103]. Homberg et al. demonstrated that LKM-1 positivity could be used to define a second type of AIH [104]. CYP2D6 demonstrates homology to HCV, herpes simplex virus, and CMV [105, 106], which initiates the molecular mimicry that triggers AIH type 2. LKM-1 staining is localized to the cytoplasm of liver cells and the P3 portion of renal tubules. However, these antibodies do not stain gastric parietal cells [105]. The LKM-1 staining pattern can sometimes be confused with the anti-mitochondrial antibody (AMA) IFL pattern [25, 107]; however, the LKM-1 staining pattern is localized to the P3 portion of the proximal renal tubules, whereas the AMA pattern is found in the distal renal tubules [108].

#### 6.2.6. LKM-3 and Other LKM Antibody Subtypes

The presence of LKM-2 antibodies has been described in patients with drug-induced hepatitis that is caused by tienilic acid (which was subsequently withdrawn from the market) [109]. 

LKM-3 antibodies have been described in patients with chronic hepatitis D [108, 110] and HCV-induced AIH [111, 112]. In addition to the LKM-1 antibodies, LKM-3 antibodies are detected in 5–19% patients with AIH type 2 [108, 112, 113]. However, LKM-3 antibodies are occasionally the only marker that can be identified in some cases of AIH [113–115]. LKM-2 and LKM-3 antibodies can both be observed within the exocrine pancreas and the thyroid by IFL [113]. LKM-3 IFL also demonstrates the staining of the adrenal gland and stomach [108]. The main molecular target of LKM-3 antibodies is uridine diphosphate glucuronyl transferase [112, 113]. A fourth type of liver microsomal antibody (anti-LM) was shown to target cytochrome P50 (CYP), CYP1A2, CYP2A6, and CYP2B6; these antibodies are detected in cases of AIH that are associated with autosomal recessive disorders, which constitute polyendocrinopathy-candidiasis and ectodermal dystrophy (APECED) [116–118]. APECED-associated AIH is currently the only known form of AIH that exhibits a Mendelian pattern of inheritance [119]. In addition, CYP1A2 may also play a role in drug-induced liver disease [115]. Anti-LMs that target CYP2A6 and CYP2B6 demonstrate an IFL pattern that is similar to that of LKM-1 antibodies [116], whereas CYP1A2 only demonstrate a liver microsomal pattern [116]. 

#### 6.2.7. Anti-Liver Cytosol Type 1 (LC-1)

The LC-1 antibodies, which target the enzyme formiminotransferase cyclodeaminase (FTCD), are present in 32% of the LKM-1-associated cases of AIH type 2 [120–122]. However, the presence of LC-1 and LKM-1 antibodies can obscure the LC-1 staining pattern [108, 123]. LC-1 antibodies are also detected in 14% of anti-LKM-1-positive chronic hepatitis C patients [123]. In addition, LC-1 antibodies also serve as a marker of liver inflammation and the rapid progression of liver disease in AIH patients [124]. 

#### 6.2.8. Anti-Asialoglycoprotein Receptor (ASGPR)

The ASGPR is a major liver-specific protein [125, 126] and a type II transmembrane glycoprotein. To date, the ASGPR is the only liver-specific antigen to be identified in AIH [108, 125–127]. Antibodies that target the ASGPR are mainly detected in patients with both types of AIH, but mainly AIH type 1 (approximately 88–90%) [128, 129]. Moreover, anti-ASGPR antibodies may represent a useful prognostic indicator. High levels of anti-ASGPR are often associated with inflammatory activity [10, 128–130]. The levels of anti-ASGPR decrease in response to immune-suppressive therapy and reappear with the relapse of AIH [127, 130]. However, the applicability of ASGPR antibody detection for the diagnosis of AIH has been limited by the lack of disease specificity and the difficulty in the development of a reliable molecular-based assay [75, 108].

#### 6.2.9. Perinuclear Antinuclear Neutrophil Cytoplasmic Antibodies (P-ANCA)

P-ANCAs are detected by IFL using a neutrophil substrate [131, 132]. A typical form of p-ANCAs has been identified in patients with AIH type 1, primary biliary cirrhosis, and primary sclerosing cholangitis [133, 134]. These antibodies target a peripheral nuclear perinuclear antigen (p-ANCA) [108, 135] and are associated with more severe forms of AIH type 1 [134, 136]. In the report published by Roozendaal et al., 51 of the patients with positive P-ANCA exhibited cirrhosis compared to the 15 P-ANCA-negative patients with liver cirrhosis [134]. Similarly, in the same report, 16 P-ANCA-positive patients compared with 5 P-ANCA-negative patients had active disease. Furthermore, the chance of relapse was higher in the P-ANCA-positive patients, as reported by Roozendaal et al. [134].

#### 6.2.10. Antibody to Histone and Double-Stranded DNA (dsDNA)

Antibodies to dsDNA have been detected in patients with AIH type 1. The presence of these antibodies has been associated with high immunoglobulin G (IgG) levels and a poor immediate response to corticosteroid treatment compared to anti-dsDNA-negative patients [137].

#### 6.2.11. Anti-Chromatin

Anti-chromatin staining is detected in 39% of the ANA-positive AIH patients and is more commonly detected in males with AIH. These antibodies are associated with high levels of IgG and active disease [138]. 

#### 6.2.12. Antimitochondrial Antibody-M2

The presence of AMA-M2 has been reported in 8–20% of typical AIH patients. Furthermore, AMA-M2-positive AIH is associated with the presence of DRB1*03, DRB1*04, and DRB1*13, the histological features of chronic active hepatitis and probable or improbable pretreatment scores [139–141]. However, compared to PBC patients, AIH patients with this form of AMA have lower AMA-M2 titers and tend to exhibit a favorable response to corticosteroids [139, 140].

### 6.3. Elevated Serum Immunoglobulin G (IgG)

The elevated levels of serum IgG and low C3- and C4-complement levels in AIH patients were first described by Thompson et al. in 1973 [114]. High levels of IgG can be found in both AIH type 1 and type 2 patients [10, 11, 36, 142, 143], although 25% of the AIH type 2 patients may demonstrate normal IgG levels [25]. In our previous report comparing serum IgG levels between AIH patients and patients with other chronic liver diseases, we found that the mean serum IgG level was 30.6 g/L in AIH patients. Moreover, this level demonstrated a sensitivity of 90–98% and a specificity of approximately 96% for the diagnosis of AIH [144].

## 7. Classification of AIH Based on Serological Markers

### 7.1. AIH Type 1

AIH type 1 is the predominant form of AIH and affects all age groups, although it is more commonly diagnosed in individuals between 20 and 40 years of age [17, 18, 21, 24]. AIH type 1 is characterized by positive staining for ANAs and/or SMAs, both of which can be detected in 80% of the patients. Both markers are non-disease specific and can be used to determine patient prognosis [25, 26, 75, 115]. SLAs, which represent the most specific autoantibody for AIH type 1, are also detected in one-third of cases [100, 101]. This form of AIH is the most commonly reported form of AIH in North America and in European and Asian countries [10, 11, 17, 18, 20, 21, 23, 24]. Occasionally, patients with AIH type 1 are negative for both ANAs and SMAs, although these antibodies may be detected at a later point during the course of the disease [145].

AIH type 2 is characterized by the detection of LKM-1 antibodies, which are directed predominantly against CYP2D6 [16]. AIH type 2 is a severe form of acute hepatitis that occurs mainly in children [108]. More than 90% of children with AIH type 2 will demonstrate antibody reactivity to LKM-1, CL-1, or both [78, 123, 146]. 

In total, 10–15% of the AIH patients are negative for ANAs, SMAs, and LKM-1 antibodies at the time of clinical presentation. However, 25% of these patients will demonstrate detectable levels at a later time during the course of the disease. An additional 20% will be positive for other nonstandard AIH markers, such as the SLA, LC-1, or P-ANCA antibodies, which will prove helpful in establishing the diagnosis of AIH [96, 97, 115, 125, 126, 135]. Patients with isolated SLA antibodies (approximately 5% of the AIH patients) have been classified as having AIH type 3 [16, 94, 145], although it has been suggested that this form of SLA-associated AIH should be considered a variant of AIH type 1 due to the similarities of this condition to the genetic, clinical, and serological findings that are associated with AIH type 1 [26, 108, 147]. Moreover, 5% of the patients demonstrate the typical features of AIH but are negative for autoantibody testing. [26, 75, 115]. These patients typically have a favorable response to immune-suppressive therapy [115, 147].

In our experience, we have diagnosed all of the adult patients in our center to have AIH type 1 based on biochemical, immunological, and histological features [18, 62]. Unpublished data from the Pediatric Gastroenterology Department in the same center showed that most of our pediatric AIH cases are of type 1 and only a few of the patients exhibit AIH type 2.

### 7.2. Clinical Presentation

AIH type 1 is the most common type and represents approximately 80% of all cases. Most patients with AIH who are younger than 18 years of age are diagnosed to have AIH type 1. In addition, there is a female predominance of 75% in these cases [25, 26]. 

The clinical presentation of AIH can be in the form of asymptomatic liver disease with abnormal liver test results [13, 15, 18, 21, 26, 29, 115] and nonspecific symptoms, such as arthralgia and fatigue [26]. A total of 26–49% of all AIH cases present as acute hepatitis [16, 18, 26, 29] that is characterized by right upper-quadrant abdominal pain, fatigue and jaundice [16, 18, 21]. The AIH type 2 patients have a higher rate of acute hepatitis compared to type 1 patients [108]. Furthermore, approximately 25–45% of these AIH type 2 patients develop a severe form of decompensated cirrhosis or fulminant liver failure [15, 16, 18, 20, 21, 24, 29, 33, 146]. AIH and advanced cirrhosis patients exhibit all the features of portal hypertension and hepatocellular dysfunction, including ascites, hepatic encephalopathy, and variceal bleeding [18, 24, 33, 148]. Fulminant liver failure is rare in the presentation of AIH [10]. Other autoimmune diseases may coexist in 20% of the AIH patients, including type 1 diabetes, thyroid disorders, systemic lupus erythematosus, rheumatoid arthritis, and primary sclerosing cholangitis [16, 18, 75, 149, 150]. 

### 7.3. Diagnosis of AIH: Tables 1 and 2

There is no single diagnostic marker for AIH. Therefore, the diagnosis is based on a combination of clinical, biochemical, immunological, and histological findings [11]. The diagnosis of AIH is based on a diagnostic scoring system that was established in 1993 by the International Autoimmune Hepatitis Group (IAIHG). This scoring system was revised in 1999 by the same group, as shown in Table 1 [36]. More recently, a simplified set of diagnostic criteria was proposed by the IAIHG in 2008, as shown in Table 2 [143]. This simplified set of criteria, which was validated by Yeoman et al. in 2009. [151], is less sensitive than the original scoring system (85% compared to 100%), although it demonstrates improved accuracy and specificity (90% compared to 73%) [115, 151, 152]. However, the simplified criteria perform less efficiently for the diagnosis of acute forms of AIH [153]. Similarly, both the original and the simplified scoring systems do not perform well in patients with AIH who also have fulminant liver failure or cholestatic disease [151, 152]. Another limitation of both the revised and the original scoring systems is that these have not been carefully evaluated in children [25]. According to the IAIHG, the diagnosis of AIH can either be probable or definite according to the score. The treatment response is graded positive in both the original and the revised AIHG scoring systems, and a posttreatment reassessment may upgrade the diagnostic score from probable to definite [10, 143]. Qiu et al. evaluated both sets of criteria in Chinese patients. These researchers found that the simplified criteria were less sensitive than the revised original criteria and diagnosed probable AIH in 90% and 100% of the patients, respectively; both scoring systems had a similar specificity of 95% and 93%, respectively [154]. However, the two criteria exhibited a similar performance in the diagnosis of definite AIH [154]. 

Initial clinical data should be collected from the patient to evaluate the history of alcohol use and the use of any hepatotoxic medication [10, 155]. Liver biomarker tests of the liver of AIH patients often reveal elevated alanine aminotransferase and aspartate aminotransferase levels in the serum (AST and ALT) compared to the parameters of cholestasis (alkaline phosphatase and gGT), which are generally normal or only slightly increased in AIH patients. However, the degree of aminotransferase elevation does not often correlate with the severity of the histological findings [36].

### 7.4. Serum Autoantibodies in AIH

The previously described autoantibodies are often not sufficient for the diagnosis of AIH because these can be detected in many other conditions and their levels can sometimes be undetectable during the course of the disease. [36, 145] Therefore, a single seronegative test or a low antibody titer is not sufficient to exclude a diagnosis of AIH, particularly in patients that are receiving immune-suppressive therapy [75]. ANA and SMA levels of 1 : 40 are considered significant for the diagnosis of AIH in adults [25, 78]. In addition, ANA and SMA antibody levels of 1 : 20 and LKM-1 antibody levels of 1/10 are sufficient for the diagnosis of AIH if other characteristic features are also present [36, 74, 146, 156]. However, the detection of low levels of serum antibodies in the absence of other features of AIH is not sufficient for the diagnosis of this disease because the sera of normal adults may have a low titer of ANA, SMA, and LKM-1 antibodies [156–158].

Elevated levels of serum IgG are included in both the revised scoring system and the simplified criteria that are used for the diagnosis of AIH [36, 143]. According to the simplified criteria, an IgG level that is 1.1-fold higher than the normal level is scored as +2 [143]. However, IgG levels can be normal in 15% and 25% of the patients with AIH type 1 and type 2, respectively [129]. In addition, AIH type 2 patients commonly have IgA deficiency [146]. 

### 7.5. Liver Biopsy

A liver biopsy is recommended for the definitive diagnosis of AIH. This test rules out features of alternative liver diseases and identifies features of AIH variants and overlapping syndromes [10, 11]. Moreover, an asymptomatic presentation of AIH does not rule out active or even advanced findings on liver histology [35].

Plasma cell infiltration involving both the portal and peripheral areas is typically observed in AIH [10, 11, 98, 159]. Furthermore, the histological hallmark of AIH consists of interface hepatitis (piecemeal necrosis), which is characterized by mononuclear and plasma cell infiltration in the portal areas expanding to the liver lobules and the disruption of peripheral hepatocytes through the erosion of the limiting plate. In addition, the ballooning and hepatic regeneration with rosette formation in the peripheral hepatocytes and the subsequent development of peripheral fibrosis are initially observed in cases of AIH [10, 11, 25]. 

The severity of the inflammatory activity can often predict the progression of the disease [160]. In addition, 40–80% of the cases will ultimately progress to cirrhosis with a poor prognosis [11, 159, 161–163]. Lobular necroinflammation and bridging necrosis, which are more prominent in cases of acute hepatitis, are also commonly present [11]. Zone 3 necrosis, which is a condition that can evolve into interface hepatitis, is occasionally observed in the liver biopsy [164]. An important feature of advanced fibrosis and cirrhosis in AIH compared to other liver diseases is that fibrosis in AIH is reversible with proper immune-suppressive therapy [26]. 

### 7.6. Differential Diagnosis

ANAs and SMAs may also be present in patients with chronic hepatitis (CH) B and C, although these SMAs often lack specificity for F-actin [156, 165, 166]. Similarly, p-ANCAs have also been detected in 5.6% of chronic hepatitis C patients [108, 156, 167], and LKM-1 antibodies can be detected in as many as 7% of CHC patients. The detection of LKM-1 antibodies in CHC patients is important because the treatment of these patients with interferon can unmask nonactive AIH [168]. Anti-LC antibodies have also been detected in some cases of CHC, but the significance of this remains undetermined [169]. ANAs and/or SMAs have also been detected in cases of chronic hepatitis D, primary sclerosing cholangitis, primary biliary cirrhosis, and drug-induced hepatitis [51, 55, 156, 170]. Furthermore, ANAs and SMAs have been detected in patients with nonalcoholic fatty liver disease (NAFLD) [156, 171], Adams et al. found that 23% of NAFLD patients were positive for ANA and/or SMA in their serum. Furthermore, these patients had a higher grade of fibrosis and stage of inflammation compared to ANA- and SMA-negative NAFLD patients [166]. ANAs and SMAs can be detected at a low titer of 1/40 in 13% and more than 31% of healthy individuals, respectively [157, 158]. ANAs can also be detected in 34% of patients with malignancy, and 26% of these patients exhibit a high titer [172]. Similarly, anti-ASGPR antibodies have been detected in cases of primary biliary cirrhosis (PBC) and in 14% of the cases of CHB and CHC with alcoholic liver disease [173, 174]. SLA antibodies have also been detected in 22% of patients with fulminant hepatic failure [175]. In addition to the serum antibodies, the clinical presentations of Wilson's disease can mimic AIH, especially in younger patients. The differentiation of Wilson's disease from AIH can be supported by the presence of a Kayser Fleischer ring and through urine and serum copper studies in patients with Wilson's disease [29, 176]. 

### 7.7. Treatment of AIH

#### 7.7.1. Who Should Be Treated? 

Serum levels of ALT and AST that are 10-fold higher than the upper boundary of the normal range or the combination of elevated ALT and AST levels with elevated levels of serum IgG or with the histological evidence of interface hepatitis are absolute indicators for treatment [10, 16, 35, 177]. If left untreated, these highly active forms of AIH exhibit 3- and 10-year mortality rates of 50% and 90%, respectively [11, 16, 178, 179]. Moreover, if these AIH cases are left untreated, patients generally have an 82% risk of progression to cirrhosis [10, 16]. Asymptomatic patients with active disease should be considered for treatment because 26–70% of these patients are likely to progress to cirrhosis [15, 180]. The presence of cirrhosis does not preclude treatment for AIH because histological features of cirrhosis are present in 30% of AIH patients at the time of clinical presentation. Moreover, these patients with cirrhosis generally display rates of treatment response and treatment failure that are similar to those of noncirrhotic patients [15, 18, 24, 181].

The indication for treatment is uncertain in patients with mild laboratory and histological abnormalities [10, 177]. If untreated, patients with mild disease generally have normal 5-year survival rates and a 10% chance of progression to cirrhosis within 10 years [178]. However, untreated patients with mild disease generally have a 10-year survival rate of 63% compared to the 98% survival rate that is observed with patients that are treated [179]. Considering the potential side effects of immune-suppressive therapy, the treatment of such patients should be individualized. 

Treatment is not indicated in patients who fail to present evidence of biochemically or histological, active disease, patients with decompensated cirrhosis that are listed for a liver transplant, and patients with established cirrhosis without inflammatory activity [10, 15, 16].

#### 7.7.2. Standard Treatment Regimens

The AIH treatment is divided into 2 phases, which consist of the induction of remission and the subsequent maintenance of the remission [10, 11, 15]. The standard treatment is based on either the use of corticosteroids alone or in combination with azathioprine (AZA), which alleviates the side effects that are associated with steroids [10, 11, 16, 29]. Both regimens are equally effective. Prednisolone and prednisone are initiated at a dose of 1 mg/kg with a maximum of 60 mg per day in prednisolone monotherapy or a maximum dose of 20–30 mg per day in combination therapy [10, 11, 15, 16]. Azathioprine is initiated at a dose of 1-2 mg/kg. After the ALT and AST levels normalize, the prednisolone dosage can be reduced by 10 mg on a weekly basis until a dose of 20 mg per day is achieved; a slower reduction of the dose is advisable after this point [11]. A strict adherence to the treatment regimen is important to achieve remission [11, 15, 16, 25]. A systematic review of randomized controlled trials on the treatment of AIH has shown that prednisolone monotherapy and combination therapy with AZA are both effective induction therapies for first-time treatment and relapses, whereas prednisolone in combination with AZA or AZA monotherapy is the superior maintenance therapy [182]. Patients with AIH type 1 typically demonstrate a better response to treatment than patients with AIH type 2 [29]. Furthermore, genetic factors may play a role in predicting the treatment response because AIH has a variable treatment response in different ethnic groups [35], which may be related to polymorphisms at the HLDA-DRB1 loci [35]. 

#### 7.7.3. Definition of Remission

The remission of previously symptomatic patients is defined as the recovery from symptoms, the normalization of biochemical markers of disease and inactive liver histology [10, 11, 15]. Remission is rarely achieved before 12 months of treatment and is more commonly achieved (in 75–80% of patients) within 2 years of immune-suppressive therapy [11, 15, 16]. AZA can then be used at a dose of 1-2 mg/kg for maintenance therapy [11, 183] either alone or in combination with low-dose prednisolone (2.5–10 mg daily) [11, 15, 16]. Complete remission is not achieved in 20% of patients [184, 185], and 9% of patients will experience treatment failure and the progression of liver disease [10, 186]. Treatment failure has been reported more in younger patients and in patients with acute presentations [186]. In our experience, treatment failure is observed more in patients with advanced cirrhosis [18]. 

The treatment of AIH with corticosteroids and AZA is accompanied by the risk of the many side effects of both drugs, as shown in Table 3 [10, 11, 16, 18, 115]. Patients with deficiencies in the thiomethylpurine transferase enzyme are at an increased risk of AZA toxicity [26, 187]. Similarly, patients with cirrhosis at the time of clinical presentation have a greater likelihood of experiencing immune-suppressive drug-related side effects [10, 15, 18].

#### 7.7.4. Treatment Withdrawal

The decision to withdraw patients from immune-suppressive therapy should not be attempted if there is a chance of relapse [35]. Because the histological remission lags approximately 3 to 6 months behind biochemical normalization, the withdrawal from immune-suppressive therapy should be performed after a liver biopsy demonstrates the remission of histological activity [10, 16, 26, 188]. Treatment can be withdrawn 3–6 months after complete remission [26], but not before 2 years of treatment, because withdrawal after less than 2 years of treatment is associated with a higher likelihood of relapse [10, 15, 16, 29]. The extended duration of immune-suppressive therapy for 4 years has been shown to be effective in the reduction of the relapse rate from 83% to 33% [189]. Treatment withdrawal should not be attempted in LKM-1-associated AIH type 2 [11, 29, 108]. The withdrawal from immune-suppressive drugs following complete remission is associated with a 60–86% chance of relapse [11, 115, 185, 186].

### 7.8. Relapse of AIH

A symptomatic, biochemical, and histological relapse of AIH may develop as the steroid dose is tapered or following withdrawal from immune-suppressive therapy [10, 16, 115, 185, 186]. The AASLD recommends retreatment for patients who experience their first relapse after treatment withdrawal using the same initial regimen; this treatment regimen should be followed by low-dose prednisolone or 2 mg/kg of AZA indefinitely [10].

Alternative treatments in cases of failure due to the initial immune-suppressive therapy and in cases of posttreatment relapse are the following.

1. A high dose of prednisolone together with 150 mg/day of AZA should be attempted prior to the use of an alternative agent that was not used with the initial therapy [10].
2. *Budesonide:* budesonide is a synthetic steroid with a high first-pass metabolism in the liver [16] that has been shown to be an effective alternative to the initial therapy for AIH. When combined with an AZA regimen, budesonide has been shown to be superior to prednisolone in terms of the induction of biochemical remission (47% compared to 18%). After 6 months, the use of budesonide is associated with a lower frequency of side effects [190]. However, budesonide is ineffective for the treatment of steroid-refractory or steroid-dependent AIH [191]. Multiple pilot studies on budesonide in previously untreated patients have shown limited side effects [16, 192, 193], which suggests that budesonide may represent a suitable replacement for prednisolone in the treatment of AIH. These findings have been confirmed in larger studies [190].
3. Deflazacort is another alternative corticosteroid that has been used in a small number of patients, although further studies examining its use for the treatment of AIH are needed [194].
4. *Mycophenolate Mofetil (MMF):* MMF is a purine antagonist that has been used as an initial treatment for refractory or relapsed AIH patients [195, 196]. Nine studies with a small number of patients (the largest study included 37 patients [197]) have investigated the use of MMF in AIH. In 8 of these studies, variable degrees of remission (39–100%) were achieved [195]. However, the use of MMF is limited by its side effects, which can be severe in some cases [195, 196]. Because MMF metabolism is independent of thiopurine methyltransferase, this drug can replace AZA for the treatment of AIH in AZA-intolerant patients [188]. Two of our patients who exhibited standard treatment failure received MMF; one of these had a complete response to MMF and the other had a partial response that was exhibited by the deterioration of biochemical parameters, which improved after MMF was discontinued [18].
5. *Cyclosporine and tacrolimus:* These drugs are calcineurin inhibitors and potent immune-suppressing agents (tacrolimus is more potent than cyclosporine). Pilot trials on cyclosporine and tacrolimus have demonstrated that these drugs are effective for the induction of remission in refractory AIH patients [16, 198–200]. However, treatment with calcineurin inhibitors is limited by the potentially severe side effects.

Other medications that have been evaluated alone or in combination with steroids in a small numbers of patients who failed to respond to standard AIH therapy include D-penicillamine, cyclophosphamide, and ursodeoxycholic acid [11, 16, 188]. Mucenic et al. studied the effect of chloroquine in 15 patients with AIH and found that chloroquine-treated patients had a lower rate of relapse [201].

### 7.9. Future Biological Treatments for AIH

Tumor necrosis factor alpha (TNF*α*) has been shown in an animal model to induce AIH [11]. When used for chronic inflammatory disorders, infliximab and adalimumab have been reported to cause drug-induced AIH [52, 53]. Recent data suggest that the anti-TNF*α* drug infliximab may be effective as an alternative treatment for AIH [202]. However, anti-B cell therapy with anti-CD20 antibodies (rituximab) has also been attempted in a small number of patients with AIH and has shown promising results [11, 203].

Tregs also play a major role in the pathogenesis of AIH, and Treg-mediated therapy is another future alternative that requires additional study [10, 11, 198]. 

Abatacept is an agent that blocks the immune system by acting on cytotoxic T cells. This drug has been approved for the treatment of rheumatoid arthritis and is another suggested future therapy for AIH [115].

### 7.10. AIH in Special Groups

In total, 2.3% of AIH patients require liver transplants, and a diagnosis of AIH is the indication for liver transplantation in 4–6% of all liver transplant patients [10, 16, 204].

Liver transplantation may be urgently required in AIH patients with severe acute refractory presentation of fulminant liver failure [11, 115, 198, 205, 206]. Liver transplantation is also indicated for patients with end-stage cirrhosis (nearly 10% of all AIH patients) [11, 16, 19, 115, 197]. The 5-year survival rate for these patients is 80–90%, and the 10-year survival rate is 75% [115, 207]. According to the European transplant database, the 5- and 10-year post transplantation survival rates are 76% and 66%, respectively [16]. Post-liver transplantation-recurrent AIH has been reported in approximately 25–30% of patients [10, 208, 209], and recurrent AIH is more common after steroid discontinuation [10, 16, 209]. HLA-type or donor mismatches may also contribute to the recurrence of AIH [16, 209]. Currently, there is no validated scoring system for the diagnosis of recurrent AIH. In addition, no clinical trials have been conducted on the treatment of post liver transplantation recurrent AIH. However, one study found that the reintroduction of steroids, together with adjustments to the dose of calcineurin, proved effective in certain patients [209]. MMF is an alternative treatment option for patients who do not respond to this approach [10]. 

## 8. *De Novo* AIH

AIH has been reported to develop after liver transplantation for other diseases [115, 209, 210] and is more frequently reported in children than adults [11]. AIH may represent a form of cellular rejection due to suboptimal immune-suppressive therapy [209, 211, 212]. De novo AIH is responsive to treatment with steroids and AZA [10, 212, 213].

## 9. Fulminant Hepatitis

Acute presentation with fulminant hepatitis has also been reported in cases of AIH [214]. Corticosteroids are effective in 36% to 100% of fulminant AIH cases [215, 216], and liver transplantation is indicated for unresponsive patients [196, 215].

## 10. AIH during Pregnancy

AIH occasionally develops during pregnancy or shortly after delivery. This AIH presentation demonstrates a favorable outcome in most patients [18, 21, 206, 215]. Pregnancy is associated with an immune-suppressive state, which may help maintain the remission of the disease during pregnancy [12, 206]. Schramm et al. reported a 26% incidence of adverse pregnancy outcomes in 42 pregnancies in AIH patients. In cases in which AIH developed during pregnancy, these researchers found that there was an increased rate of fetal mortality but not maternal mortality [217]. Furthermore, the adverse events were highly associated with the presence of SLA [217]. However, there are no data on the safety of AZA for the treatment of AIH during pregnancy [215], although steroids and AZA have generally been found to be relatively safe [11, 206], which suggests that this treatment should be continued during pregnancy (preferably with corticosteroids alone) [215]. MMF has been shown to have teratogenic effects in an animal model, and this drug should therefore not be used during pregnancy [196]. AIH frequently relapses during the postpartum period, which suggests that close monitoring of these patients is warranted [198, 215]. 

## 11. AIH in Elderly Patients

Approximately 20% of all cases of AIH develop in patients over 60 years of age [31, 32, 219]. The diagnosis of AIH in the elderly can be affected by the presence of comorbid disease or the use of medications for chronic medical diseases [206]. In some reports, elderly patients were more likely to have advanced disease at the time of clinical presentation [32, 218]. However, these patients tend to exhibit a favorable response to treatment. [206, 215]. The diagnostic criteria for AIH in the elderly are similar to the criteria used for younger patients. The immune suppressive treatments for AIH in the elderly demonstrate a rate of remission that is similar to that obtained with younger patients and a lower rate of relapse [32, 219]. An additional bone maintenance regimen should be included with the corticosteroid treatment of elderly AIH patients [215].

## 12. Syndromes Overlapping with AIH

AIH may coexist with the biochemical, radiological and histological features of other forms of autoimmune liver disease, namely, PBC and PSC [220, 221].

An AIH-PBC overlap is the most commonly recognized form [221]. A diagnostic scoring system for the AIH-PBC overlap was established by Chaz et al. [222]. 

The AIH-PSC overlap is less recognized compared to the AIH-PBC overlap and is more common in children than adults [223]. Patients with an AIH-PSC overlap may demonstrate the typical radiological features of PSC [224]. The treatment of AIH-overlapping syndromes is empirical and depends on the predominant biochemical, immunological and histological features. Moreover, the standard immune-suppressive therapy in combination with ursodeoxycholic acid is recommended for patients with overlapping AIH and PBC or PSC [215, 221]. Compared to AIH alone, both of these conditions, although particularly the AIH-PAS overlap, are associated with a worse prognosis and a reduced treatment response [215, 220]. In our experience, two patients with biochemical features of cholestasis (one had liver biopsy consistent with AIH and the second had immunological markers consistent with AIH type 1 but liver biopsy features of cholestasis) failed to respond to immune-suppressive therapy and were thus referred for liver transplant [220]. However, our other patients who exhibited cholestasis and the typical immunological and histological features of AIH had favorable initial responses and sustained remission as a consequence of immune-suppressive therapy [18]. 

## 13. Hepatocellular Carcinoma in HCC in AIH

HCC is much less commonly reported in AIH patients compared to other causes of liver cirrhosis [10–226]. The surveillance for HCC in AIH patients with cirrhosis is recommended and should be performed in a similar manner as with other liver diseases [10].

## Figures and Tables

**Table 1:** Revised scoring system for the diagnosis of autoimmune hepatitis.

Parameters/features	Score	Notes*
Female sex	+2	
ALP : AST (or ALT) ratio:		
<1.5	+2	1
1.5–3.0	0	
>3.0	−2	
Serum globulins or IgG above normal		
>2.0	+3	
1.5–2.0	+2	
1.0–1.5	+1	
<1.0	0	
ANA, SMA, or LKM-1		
>1 : 80	+3	2
1 : 80	+2	
1 : 40	+1	
<1 : 40	0	
AMA positive	−4	
Hepatitis viral markers:		
Positive	−3	3
Negative	+3	
Drug history:		
Positive	−4	4
Negative	+1	
Average alcohol intake		
<25 g/day	+2	
>60 g/day	−2	
Liver histology:		
Interface hepatitis	+3	
Predominantly lymphoplasmacytic infiltrate	+1	
Rosetting of liver cells	+1	
None of the above	−5	
Biliary changes	−3	5
Other changes	−3	6
Other autoimmune disease(s)	+2	7
Optional additional parameters:		8
Seropositivity for other defined autoantibodies	+2	9
HLA DR3 or DR4	+1	10
Response to therapy:		
Complete	+2	11
Relapse	+3	
Interpretation of aggregate scores:		
*Pretreatment*:		
Definite AIH	>15	
Probable AIH	10–15	
*Posttreatment*:		
Definite AIH	>17	12
Probable AIH	12–17	

*[36].

**Table 2:** Simplified diagnostic criteria for autoimmune hepatitis.

Variable	Cutoff	Points
ANA or SMA	≥1 : 40	1
ANA or SMA	≥1 : 80	
or LKM	≥1 : 40	2*
or SLA	Positive	
IgG	>Upper normal limit	1
	>1.10 times upper normal limit	2
Liver histology (evidence of hepatitis is a necessary condition)	Compatible with AIH	1
	Typical AIH	2
Absence of viral hepatitis	Yes	2
		≥6: probable AIH
		≥7: definite AIH

**Table 3:** Common side effects of initial immune suppression therapy.

(A) Side effects of prednisone/prednisolone	
(i) Acne	
(ii) Moon-shaped face	
(iii) Striae	
(iv) Dorsal hump	
(v) Obesity	
(vi) Weight gain	
(vii) Loss of bone density (bone density testing is required with bone density treatment in elderly)	
(viii) Diabetes mellitus (especially in elderly)	
(ix) Adrenal suppression on long use	
(x) Cataract	
(xi) Hypertension	

(B) Side effects of azathioprine	
(i) Nausea	
(ii) Vomiting	
(iii) Abdominal discomforts	
(iv) Hepatotoxicity (sometimes confuse with the flare of AIH)	
(v) Rash	
(vi) Bone marrow suppression with leukocytopenia (may require dose readjustment, more common in cases of cirrhosis)	
(vii) Risk of lymphoma	
